# Gendered vulnerabilities and grassroots adaptation initiatives in home gardens and small orchards in Northwest Mexico

**DOI:** 10.1007/s13280-016-0832-3

**Published:** 2016-11-22

**Authors:** Stephanie Buechler

**Affiliations:** School of Geography and Development and Udall Center for Studies in Public Policy, University of Arizona, 2323 E. Mitchell Street, Tucson, AZ 85719 USA

**Keywords:** Adaptation, Climate change, Feminist political ecology, Home gardens, Orchards, Water

## Abstract

With the retreat of the state under neoliberalism, the lack of (or negligible) government and non-governmental support reasserts grassroots initiatives as a global-change strategy. A feminist political ecology approach and the concept of adverse inclusion were used to facilitate an analysis of social differences shaping local-level adaptive responses. Adaptive responses of small farmers in the border village of San Ignacio, Sonora, Mexico, who are increasingly vulnerable to climate change, water scarcity, and changing labor markets were studied. Gender differences in production sites translate into diverse vulnerabilities and adaptive strategies. Local capacities and initiatives should be a focus of research and policy to avoid viewing women and men as passive in the face of global change. The dynamic strategies of San Ignacio women and men in home gardens and small orchards hold lessons for other regions particularly related to adaptation to climate change via agrobiodiversity, water resource management, and diversified agricultural livelihoods.

## Introduction

Northwest Mexico is a region hard hit by climate change, with frequent and prolonged drought affecting agricultural livelihoods. With the retreat of the state in Mexico under neoliberal regimes, the lack of (or negligible) government and non-governmental support for small-scale agriculture reasserts grassroots initiatives as a critical global-change adaptive strategy. In this article, I conceptualize human adaptation to environmental change as physical, social, and political “adjustment[s] in…human systems in response to actual or expected climatic [or other environmental] stimuli or effects, which moderate harm or exploit beneficial opportunities” (Dankelman 2010, p. 7). The concept of adaptation implies a longer timeframe than more immediate coping strategies. Coping strategies are short-term changes in behavior to address immediate crises; however, they may not be sustainable or they may be ineffective in addressing long-term environmental challenges to livelihood security (Pelling 2011).

Using a feminist political ecology (FPE) framework, this study focuses on micro-level adaptation in agriculture in the village of San Ignacio, Sonora, in Northwest Mexico, and offers new sites for FPE analysis: home gardens and small orchards. FPE facilitates the examination of social difference shaping vulnerabilities as well as adaptive responses at the micro (household and individual) level. In so doing, FPE elucidates gendered livelihood strategies, access to (or exclusion from) formal programs aimed to enhance adaptive capacity, such as agricultural extension and climate services, and social differentiation in agrarian power dynamics. Adaptation strategies in San Ignacio vary by gender mainly because women and men cultivate different kinds of land. Strategies have been largely grassroots in nature due to the very low levels of government support for intensive fruit and vegetable production in general and particularly production on small parcels of land—these typify women’s production niches in the study area. This research contributes to adaptation literature on both agriculture in Mexico (see e.g., Liverman 1999; Eakin 2006; Tucker et al. 2010; Mercer et al. 2012; Scott and Buechler 2013) and gender and agriculture in Mexico (Buechler 2009; Jungehülsing 2010; Martínez-Corona 2012; Bee 2013), addressing influences of landholding size and location as well as access to water, credit, and time invested on adaptation processes. This study adds to Bee’s adaptation research on women’s work in and around the home and the space of the *solar*—or home compound (Bee 2013); to date, this space has been understudied in adaptation literature despite its critical importance for women’s livelihoods. Production spaces like home gardens or small orchards have rarely been the focus of literature on adaptation; such spaces form the focus of this research.

## Background

### Feminist political ecology (FPE) framework

Women’s and men’s strategies in San Ignacio to counter their multi-faceted vulnerabilities suggest the need to rethink adaptation analyses which often overemphasize the role of outside ‘experts’ in developing solutions to future climate change threats (van Aalst et al. 2008). This study of San Ignacio combines a focus on adaptation with FPE that provides a framework for the study of “how the same dynamics that produce unequal access to resources or disproportionate vulnerabilities to environmental change are often key components of social and political difference” (Hanson and Buechler 2015, p. 8). As Rocheleau et al. argued in their early work on FPE, gender should be considered a “critical variable in shaping access and control, interacting with class, caste, race, culture, and ethnicity to shape processes of ecological change, the struggles of women and men to sustain ecologically viable livelihoods, and the prospects of any community for sustainable development” (1996, p. 4).

The nature of women’s and men’s environmental, social, economic, and political vulnerability in San Ignacio exemplifies the concept of adverse inclusion in which the poor are not incorporated as equal partners into organizations and institutions related to land, water, labor, and credit and other economic assistance that exert control over their livelihoods. Taylor (2013) has linked this concept to adaptation to climate change by examining how power brokers affect vulnerabilities and adaptation capabilities. In this study on San Ignacio, the roles of government agencies, larger landholders, employers, and household members in mediating access to social, economic, and environmental resources are assessed. Adaptation literature has underplayed the importance of the need to better coordinate government agencies and non-profits’ programs and projects with locally developed adaptation initiatives (Baas and Ricoy 2014). Examples of broader efforts involving outside agencies are discussed here.

I utilize the concept of adverse inclusion to examine local vulnerabilities and community-level adaptation in San Ignacio as well as policy options that build on community initiatives. However, I also take into account the larger policy context such as government agrarian support and policy. As Rocheleau and Roth (2007) have argued, actors’ strategies to deal with global environmental change entail interactions between institutional, political, and economic processes. Intra-community differences in institutional, political, and economic processes are clearly evident in access to both tangible (for example, land, water, financial capital) and intangible (information, time, social capital) assets—and this differential access affected actors’ capacities related to adaptation. The combined FPE and adverse inclusion conceptual approach followed here focuses on how gender and social class affect access to assets, especially land and water resources. FPE also helped reveal how women’s and men’s different roles and responsibilities produced unique knowledge and stimulated different adaptive responses in San Ignacio. Scalar issues emerged from an analysis that started in and around the home, which continues to be an important locus for women in rural Mexico, and indeed, for women globally (Rocheleau et al. 1996)—and extended to community and regional levels. Home gardens are important components of living spaces that include the living quarters as well as the land around the home. Both the home and land around the home are assets women inherit and exert control over.

### Study area

**Fig. 1:**
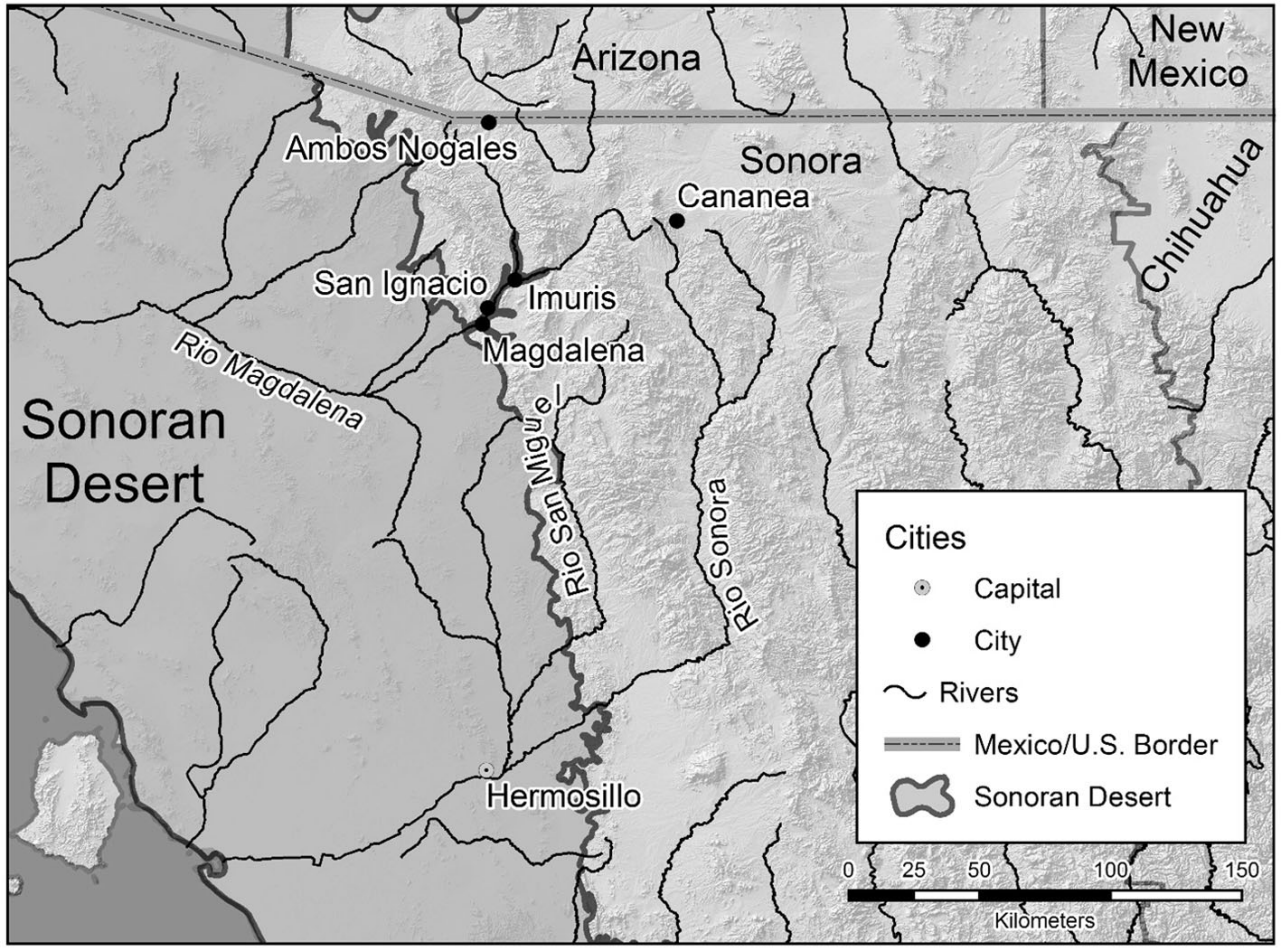
Location of San Ignacio within Sonora. *Source*: Cartographic Design, Gary Christopherson, School of Geography and Development. University of Arizona

San Ignacio, Sonora, is a rural town of approximately 1140 people (INEGI 2010a) near the small city of Magdalena with a population of 26 605 in 2010 (INEGI 2010b) and about 89 km from the Arizona–Mexico border (Fig. 1). Proximity to the border, high migration rates (especially to Arizona and California), and high in-migration from the border city of Nogales with a population in 2010 of 212 533 (INEGI 2010b) and other areas in Sonora and other states in Mexico, all contribute to shaping women’s and men’s worldviews in San Ignacio. They are accustomed to combining work from farm and non-farm sources with migration to the United States. Tourists from the U.S. and Mexico visit San Ignacio with its mission church and other historic buildings, and this provides some income mainly in the form of the purchase of processed products as well as meals.

Around 1693, Jesuit priests brought fruit-grafting methods to indigenous peoples such as the Papago and Pima when they developed the missions in San Ignacio and surrounding areas; thus farmers there have a long history of producing specialized fruit including quince (*Cydonia oblonga*), pomegranate (*Punica granatum*), and figs (*Ficus carica*) (Miller 1981; Nabhan et al. 2011). Gradually, farmers in San Ignacio added fruits such as citrus (sweet limes, lemons, grapefruit, and oranges), many varieties of plums (*Prunus domestica, P. salicina*), peaches (*Prunus persica*), pears (*Pyrus communis*), guavas (*Psidium guajava*), olives (*Olea europaea*), and persimmons (*Diospyros kaki*) and sold them to neighbors and buyers from Magdalena and Imuris, Sonora, and other nearby cities. Wholesalers also began to purchase the produce, selling it from trucks across the border in Tucson and other cities in Arizona. Since 2009, the area devoted to agricultural production has rapidly diminished due to land sales for the construction of homes for people who work in Magdalena and other cities.

San Ignacio sits at the edge of the Sonoran desert, which is experiencing the most pronounced winter and summer warming trends of the border (Weiss and Overpeck 2005; Wilder et al. 2013). With climate change, increased El Niño activity is producing a northward shift of the polar jet stream, translating into greater temperature variability including periods of unusually cold temperatures (Lankao and Smith 2014) for locations like San Ignacio. Lower winter, spring, and summer rainfall have been documented for the entire border region with increased drought (Wilder et al. 2013), negatively impacting farming-based livelihoods (CLIMAS 2013; Serrat-Capdevila et al. 2013). This intensifies vulnerabilities particularly among populations directly dependent on natural resources. Water resources are under extreme pressure in the Concepción River basin and along its tributary, the Magdalena River. San Ignacio is divided by the Magdalena River that flows a short distance from the main plaza. Two dams and wells in this river supply water to Nogales and Magdalena cities, straining rural water supplies (Prichard and Scott 2013). Water levels in the springs and wells that irrigate orchards and fields in San Ignacio also vary according to rainfall-dependent flows in the Magdalena River. Agriculture practiced in home gardens is often irrigated with water from a community well; this well had to be deepened recently.

## Materials and methods

In this ongoing study, I have conducted 70 in-depth interviews of 40 women and 30 men and 35 follow-up interviews in San Ignacio, Sonora from October 2007 to March 2016. All interviewees’ names included here are pseudonyms. To obtain a stratified sample of households with access to different landholding sizes, I consulted San Ignacio’s *ejido* president, the spring water irrigation user association president, a fruit and vegetable farmer, and a fruit processor. The processor assisted me with the selection of women who cultivate different crops in their home compounds and engage in different kinds of processing activities. These two presidents (of the *ejido* and the water user association), the farmer, and the processor provided tours of critical water sources in the community, showed me several home gardens and orchards, and invited me to community events. Interviews addressed the production and processing of fruit and vegetables, each household member’s participation in these activities and other livelihood activities, any changes in climate and water they have experienced over the past 20 years that have impacted agriculture and their responses to these changes. While conducting the research, I stayed in an agricultural processor’s home, providing me invaluable insight into community dynamics and agricultural production. Studying San Ignacio over several years led to a deeper understanding of the complex effects changing climate, water, and economic conditions as well as a lack of government assistance has had on these actors and their largely grassroots adaptation activities.

## Results and discussion

### Agriculture in Sonora state

Sonora is among the top six agricultural producers and second in irrigated area within Mexico (FAO 2013). Approximately 11.2 % of Sonora’s economically active population is directly employed in agriculture (SAGARPA 2011). Major feed crops (alfalfa, sorghum, rye grass, feed corn) were cultivated on 53 486 hectares, whereas fruit was cultivated on 1411 hectares and vegetables on 40 692 hectares in 2009 in Sonora (OEIDRUS 2010a–f). A rising percentage of Sonora’s government assistance is for non-food crop production, mainly forage for animals such as alfalfa, a water-intensive crop nevertheless subsidized by the Secretariat of Agriculture, Livestock, Rural Development, Fisheries and Food (SAGARPA) (INEGI 2015).

Extension services for producers (especially related to irrigated crops) were decimated throughout Mexico since the government agency related to agriculture and water was divided into two separate agencies titled SAGARPA and CONAGUA beginning in 1976, and neoliberal agricultural reforms initiated in 1986 with Mexico’s acceptance of the General Agreement on Tariffs and Trade (GATT), and the passing of the North American Free Trade Agreement (NAFTA) in 1994. These reforms, which emphasized the opening of the market to foreign trade and a reduction in the direct involvement of the state in agriculture, shaped the context for a reduction in SAGARPA’s budget over time with only a slight recuperation of these cuts in 2015 (Hernández-Rodríguez 2013; SAGARPA 2014). Government programs have not improved adaptive capacities of food producers in part by aggravating existing challenges related to water availability.

### Production in San Ignacio

Small spaces or plots of land are often used for fruit and vegetable production in San Ignacio. Women predominate in farming home gardens in their *solar* or home compound—a space they inhabit more than men. Two of the main factors that shape this practice are (a) gender roles that continue to place childcare and housework responsibilities primarily on women thereby tying them more to the home and its environs and (b) inheritance patterns that generally allocate the home and land around it to women (and provide men with larger agricultural plots away from the home). FPE research has emphasized the importance of making explicit gender differences in access to and control over natural resources, and how these differences shape prospects of developing sustainable livelihoods (Elmhirst 2015; Buechler 2015). By growing crops and making preserves for home consumption and for sale, women contribute to the household economy and have access to income that they are able to control. Their ability to produce livelihoods from these home gardens has been impacted by environmental conditions. Their crops are affected by climate change, and water supply is often insufficient. However, to date, these smaller plots of agricultural land have been seemingly invisible to agriculture and water-related agencies. In part, this lack of attention is due to persisting, widely held beliefs (in Mexico and globally) in a false dichotomy between reproduction and production which categorizes spaces in and around the home as arenas for reproductive activities only.

**Fig. 2:**
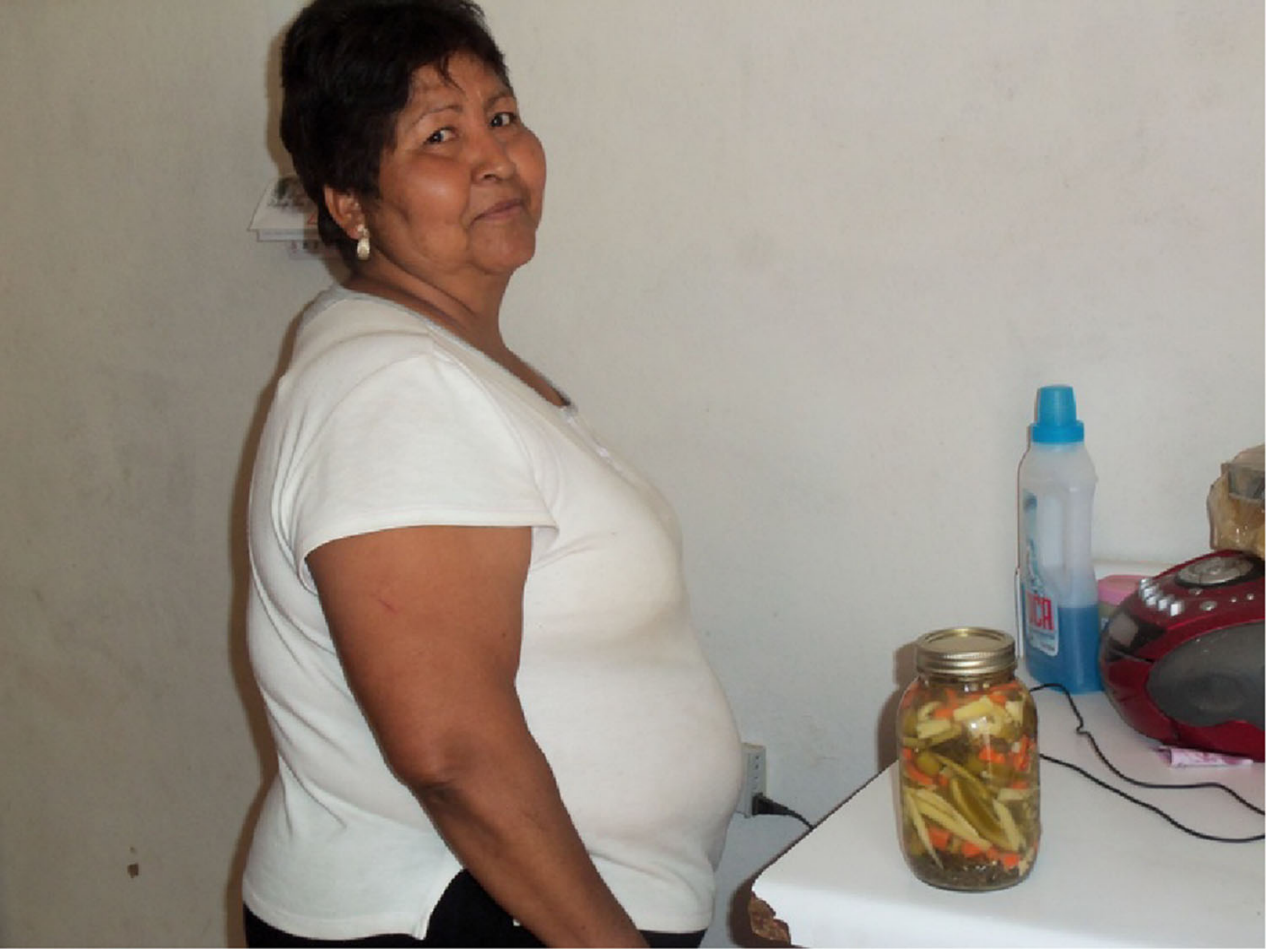
Home gardener selling her pickled vegetables from her house. Photo credit: Stephanie Buechler

In addition to small-scale agriculture, the home compound in San Ignacio serves as the main location for food processing-related work; crops are converted into quince paste and jam, fig jam, canned quince, peaches and pears, candied lemons and fruit popsicles as well as lye-cured and salted olives, olive oil, and pickled vegetables (Fig. 2). Skills related to processing are passed down from generation to generation. Mainly women are involved in the processing stages in the activities with the exception of quince paste where men are involved in the beginning stages of grinding the quince and cooking large copper vats of ground quince over a fire or gas burners.

Men (especially the oldest son) inherit larger, more valuable agricultural plots of land usually located further away from their home; they are also more likely to receive some government support, though this is also minimal. Most men’s plots consist of irrigated land ranging from 0.5 to 4 hectares and averaging 2 hectares in size. Women and children join men in harvesting fruit and vegetables on this land. Men also often hire labor for planting, irrigating, pesticide application, and harvesting, and they control income generated from this land. This income is greater than that generated from home gardens because of the larger size of these holdings.

#### Government support of producers in San Ignacio

Government support for farmers facing environmental change in Sonora is limited and, as discussed above, has been declining over time. When support is available, it is usually as cash transfers to help offset adverse environmental events. Such payments do help, albeit to a limited degree, to cushion the effects of environmental crises but do little to enhance innovation that would enable farmers to become more resilient to environmental and economic change. The majority of farmers in Sonora own a maximum of seven hectares of land; as mentioned in San Ignacio the average is two hectares. Small landowners, including those in San Ignacio, have not been very successful in obtaining government assistance for agricultural lands, and their PROCAMPO payments (federal government payments to small farmers) were frequently “lost” in local government coffers. This problem was partially remedied in 2013 when PROCAMPO began to make payments directly to farmers’ bank accounts instead of through local government offices. However, PROCAMPO benefits are available only to those with titled landholdings (the young, women, and those with insufficient resources to go through the titling procedures tend not to have land titles). Access to government funding remains a challenge, as the following example of an experience with the spring water irrigation association had revealed. The president of the irrigation user association attempted to obtain government funding to line the spring water canal with cement; he wished to increase the members’ abilities to adapt to reduced flows induced by drought, increasing temperatures and evaporation rates, and rising upstream water use by reducing the amount of channeled spring water that infiltrated into the ground via the earthen, unlined canals. His efforts were thwarted by high cost and travel time to state capital government offices to register land along the canals through the government titling program PROCEDE, which is a prerequisite for government assistance. The association members and other farmers in San Ignacio also lost a government-supported revolving loan fund in early 2007 when the few spring water user association members and other farmers in San Ignacio who are larger landowners (cattle ranchers) failed to repay their larger loans. Access to credit through revolving loans was important to farmers because other forms of government assistance to small farmers were reduced beginning in the 1980 s and because a greater share of the still available assistance was given to non-food crop producers (such as alfalfa producers) in Sonora. These examples underscore that the social position of small farmers affects their climate-related vulnerability; the concept of adverse inclusion is highly relevant to this case. In San Ignacio, large farmers caused smaller farmers to lose access to government assistance and costs were prohibitively high to comply with government requirements necessary to access assistance. This coincided with rising climate stressors such as greater variability in temperatures, storm, wind, rainfall patterns, and exacerbating vulnerabilities.

Access to such assistance is also gendered. Women and men age 65 and older who meet income guidelines (and are not enrolled in a contributory pension plan) are eligible for a social program that is a non-contributory pension plan for the elderly administered by the Federal Ministry of Social Development (SEDESOL). The Prospera program is a conditional cash transfer program that has helped women gain access to healthcare for themselves and their family members and to education for their school-age children. The amounts received for both SEDESOL and Prospera are modest, thus they do not facilitate any significant investments in household economic activities. Women have not received any government assistance for agriculture in home compounds or for processed goods produced there, demonstrating a large degree of social exclusion of this social group from the larger agricultural economy. Studies of home gardens, and women’s preponderance in cultivating these gardens (Moreno-Black et al. 1996; Keys 1999; Christie 2004, 2008; WinklerPrins and Souza 2005; Lope-Alzina 2007; Murrieta and WinklerPrins 2009; Galhena et al. 2013; Behera et al. 2016), have been brought to light, for example, their importance for household food security, for agrobiodiversity, for maintaining family ties, and for women’s income. However, they have rarely focused on governmental or non-governmental support of either the agricultural production or processing activities in these spaces. The FPE framework for this study draws attention to these issues by helping reveal gendered subjectivities related to access to natural resources in these home gardens often cultivated by women and gendered subjectivities related to policymaking for these largely invisible spaces (Rocheleau et al. 1996; Buechler et al. 2015).

#### Global environmental change effects evident to San Ignacio farmers and processors

Women and men in San Ignacio who practice agriculture and process their crops are noticing changes in climate and water availability. As discussed above, lower rainfall has been documented for the entire border region (Wilder et al. 2013). Farmers in San Ignacio reported that less water for irrigation was available especially late in the growing season (summer and fall) when fruit sets; this is leading to effects such as smaller fruit and lower yields. In Mexico, as a whole, it has been documented that farmers with access to groundwater are better positioned to withstand climate-related water resource pressures (Scott 2011). In San Ignacio, it is men in orchards with access to groundwater who are better able to deal with less rainfall (although pumping costs are rising as water table levels drop) than women in kitchen gardens. Women must generally depend on domestic water supply (from a village well reserved for domestic uses) that is in tighter supply during drier periods of the year due to shared use; a minority gains access to a neighbor’s well for irrigation. Men and women who raise trees in nurseries (small-scale operations that supply their own orchards and/or are for sale to others) attributed the higher sapling die-off rate to higher temperatures. They also noticed that fruit trees flower and bear fruit earlier in the season than before, and then stop producing fruit earlier in the season. Kimball et al. (2007) describe a controlled experiment on bitter orange trees, indicating fruit trees may bear more fruit as carbon dioxide levels rise with climate change. However, fruit producers in San Ignacio have not experienced such a benefit, perhaps because of the decline in irrigation water. To add to their woes, climate change has also increased pest attacks on trees, particularly olive trees that are less able to withstand these attacks due to long-term drought. The adaptation strategies discussed below are an attempt by male and female farmers to deal with these and other climate and water-related challenges.

### Innovative adaption strategies in home gardens

In home compounds, women grow a diverse array of fruits such as citrus, peaches, quince, pears, and figs; they also grow olives. Four different varieties of plums were produced regionally in orchards and in home gardens but all were wiped out around the year 2000 due to pests in the form of nematode infections of the roots and to drought that generally weakened the trees ahead of the infection (Buechler 2012). As an adaptive strategy, the women increased quince production and processing. Then, in 2011, women began purchasing plum saplings from nurseries outside San Ignacio and planting one or two of these young plum trees in their home gardens. Some male fruit producers doubted women could get the trees to thrive again in the area but were nevertheless interested in the experiment’s outcome. José, a 65-year-old farmer with a very varied orchard on 0.5 ha of land, who also produces fruit tree saplings commercially in his orchard expressed his doubts to me in spring 2013: “people come looking for plum trees but I do not sell them; they will not take hold so why fool people?” In August 2013, the first crop of plums was harvested from these trees, albeit in small numbers. From 2014 to 2015, plum harvests increased appreciably. As a result of the experiment’s success, plum trees are beginning to be planted again in larger orchards by (mainly male) landowners. This experiment ensures that a wider variety of crops are produced in San Ignacio. Women’s important experimentation and promotion of biodiversity in home compound gardens and the spillover effects on larger agricultural fields have been documented in other countries (see Moreno-Black et al. 1996; WinklerPrins and Souza 2005) and in the Yucatán and Bajío regions in Mexico (Lope-Alzina 2007; Chambers and Momsen 2007; Cámara–Córdova 2012). With concerns over increasing crop homogeneity, and associated implications for food security with climate change (Khoury et al. 2014), such experimentation should be recognized as an important policy area in adaptation. In San Ignacio, women engaged in micro-level adaptive responses over time, providing evidence of longer term approaches rather than short-term coping strategies. Women’s unique role in developing such responses underscores FPE understandings of gender-differentiated adaptation strategies.

Supplying irrigation water for home gardens is equally as challenging (or more so) as obtaining sufficient water for irrigation of larger orchards managed by men located further from the homes. Rosa, who has spent her entire 70 years in San Ignacio, experiments in her small home compound garden irrigated with tap water. Rosa recently started to grow hot chili peppers, green beans, and cilantro for the pickled vegetables she produces. Rosa also produces and sells jelly made from quince trees in San Ignacio. Her husband Francisco sells these processed products in nearby towns. The sale of products from the homes is undertaken by women; while most products are also marketed outside San Ignacio, this marketing is done primarily by men because it entails travel to areas away from the private domain of the household.

Rosa planted olive trees in their small home compound to see if she could grow her own olives to produce olive oil and salted, lye-cured olives to sell. She used to purchase the uncured olives as inputs for these products. She also runs a small tree nursery in this compound. Rosa irrigates her crops and trees with water from a home rooftop tank that she uses to store municipal drinking water, representing the multiple uses put on water supplied for domestic use. The well for San Ignacio’s drinking water does not have sufficient water in the late spring and summer months even though it is a deeper well drilled in early 2013. To compound Rosa’s problems, climate change related temperature variability translated into a harsh winter in 2013, causing the tank’s water to freeze and leaks to form. Rosa bought water from her neighbor with a well, then stored the water in a barrel and irrigated the crops with buckets. However, the drought, prolonged periods with higher than normal temperatures in the summer of 2013, and the lack of sufficient irrigation water killed all of the chili peppers and green beans that Rosa planted and the tree sapling that Francisco planted. In September, they replanted these crops. Rosa said they will now store water in the rooftop tank only in the warmer months of the year. For over 50 years, they had used this tank, and the water had not frozen, another indication of climate variability and local adaptation. Rosa’s decision to only store water in the warm months is evidence that she is aware of these changes and how these changes are affecting her ability to store and manage water. The division of labor between women and men in Rosa and Francisco’s case is true of home gardens in general in San Ignacio—where young saplings are raised in nurseries for replanting it is mainly men who produce these saplings and it is women who grow other crops and care for young trees, once they are replanted from the nurseries.

**Fig. 3:**
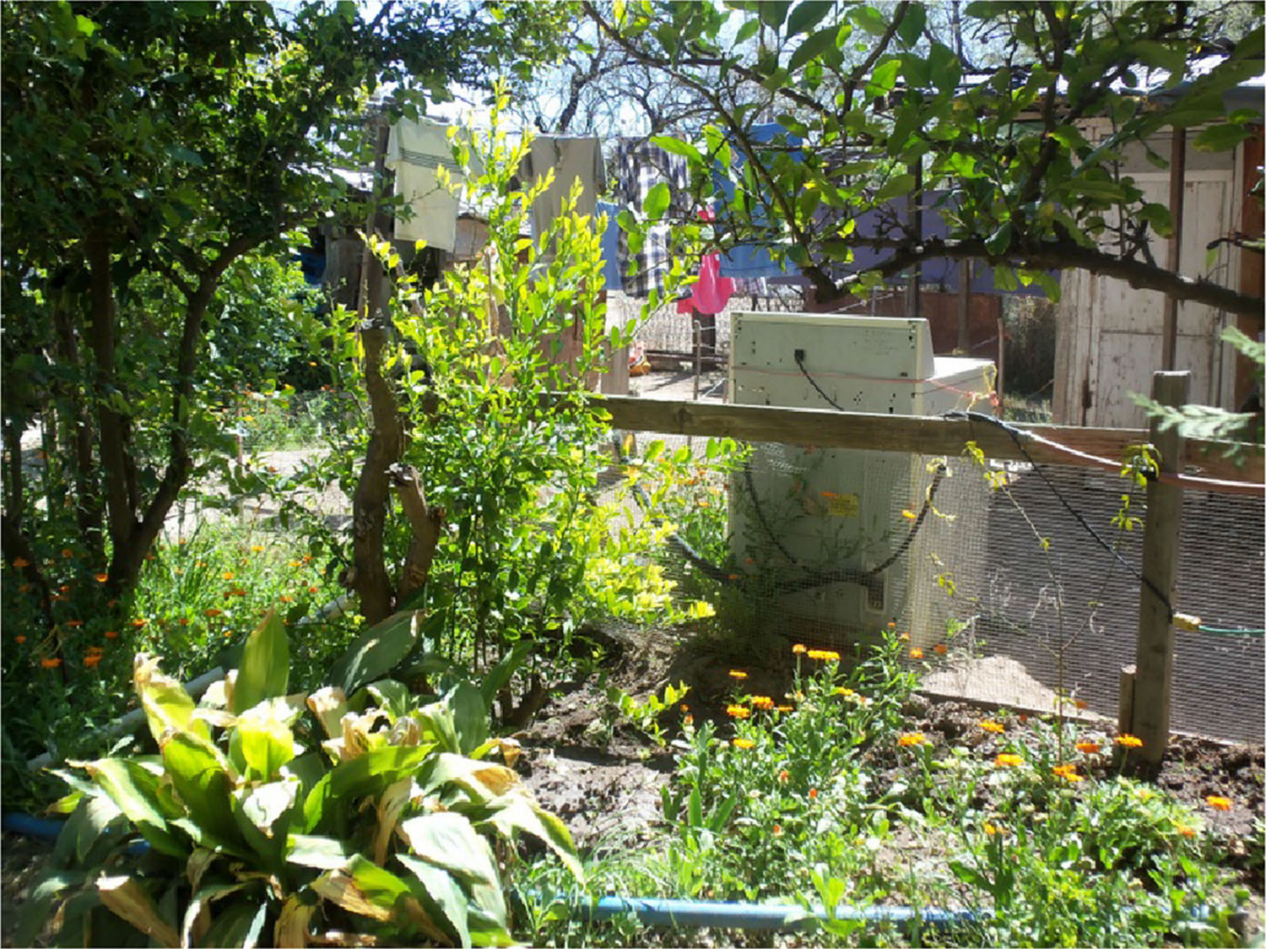
Citrus trees in home garden irrigated with greywater from washing machine. Photo credit: Stephanie Buechler

In another home garden, the household’s women irrigate tree and other crops with tap water and greywater from their laundry machine and kitchen sink in order to stretch scarce water resources (Fig. 3). Angela, a woman residing there full-time, used to sell roses and other flowers from her home compound, but these too froze in January 2013. She is not able to sell many anymore; with the lingering recession that was still affecting employment in early 2016, there was not enough cash in circulation in San Ignacio or Magdalena for most to afford such luxuries. She thus gives some flowers away to friends and neighbors and supplies the church with flowers. Angela now sells citrus flowers she collects from lemon, grapefruit, and orange trees in her home compound. She dries these citrus flowers and sells each type separately as medicinal teas. Work related to medicinal products is conducted only by women in home gardens and can be viewed as an extension of their labor as caregivers to family members.

Women’s strategies in their home compounds are dynamic, closely matched to climate and water changes they see occurring. The close proximity of these production spaces to living quarters enables them to keep a close eye on environmental changes affecting crop health. Production here is also used to gauge crop success for larger production spaces like orchards. Salleh (2009) and other feminist political ecologists have similarly noted that women’s roles bring them into daily, close contact with ecological change. Women’s strategies in home compounds such as the re-introduction and diversification of crops can be used in areas facing similar environmental challenges. Women’s agricultural production in home gardens, however, exists within a context of a lack of national government support for this gendered space as a site of agricultural production; instead the home compound is viewed merely as a site of social reproduction and even this is supported only minimally as mentioned above. Women’s agricultural production also exists in a state-level context within Sonora of negligible support for fruit and vegetable production.

### Adaptation strategies on small agricultural plots

A household with a 0.5 ha orchard located a 15 min walk from home is headed by José, referred to above. This orchard has the largest array of fruit trees in the community. He and his nephew provide the main labor for this plot. Larger plots of agricultural land are predominantly male domains in San Ignacio in terms of labor and ownership. The female members of most households tend their home gardens and process the fruits (see case of Angela); home gardens are much smaller plots of land. The orchard run by José is irrigated with spring water in a canal. The water levels in the spring are directly dependent on rainfall and on water use upstream in the Magdalena river basin. In addition to many different types of citrus fruit, peaches, pears, quince, pomegranates, and figs, the land is planted with chilies and nopal cactus (also known as prickly pear cactus from the genus *Opuntia*). José explained that “javelinas [wild pigs, *Pecari tajacu*] eat my cactus so what I do is I keep one row in front for them; the rows behind are for me to harvest as the leaves become ready to harvest…the price has been very good for nopal.” Javelinas have been affected by loss of habitat due to human encroachment; this farmer’s strategy reveals that he understands the delicate ecological balance between humans and animals. His strategy requires minimal labor and monetary costs and no harmful measures against the javelinas.

Nopal cactus requires little to no irrigation and withstands high temperatures, is nutritious and in demand especially for pickled nopal nationally and in the U.S. In San Ignacio, and across Mexico, it is women who process and pickle the nopal in their homes. Despite this, government or non-governmental programs related to agriculture do not offer support for nopal production (a mainly male task) or processing (a mainly female task) even though promoting this crop could reduce water and temperature-related vulnerability. An FPE approach was used to study female farmers in Egypt with land along the Nile River who prefer growing nopal that, like in Sonora, is ideally suited to arid conditions and requires minimal labor inputs. In Egypt, as in Sonora, Mexico, there is a lack of government support for nopal cactus production (Najjar 2015).

The farming household discussed above (the orchard run by José) lost their limas and royal lemons in January 2013 freeze. Angela and Rita, José’s sisters, cook and can about a third of this orchard’s fruits. By September 2013, two different types of quince, four types of pomegranate (white to dark red), pear, peach, and persimmon production were bountiful, and they were able to sell enough fresh fruit from their home and door-to-door in Magdalena and process enough fruit to pay off medical-related debts. The variety of fruit produced allows this farming household to recover more quickly from climate-induced damage and economic change. If one type becomes affected by extreme weather events, other types that ripen in other seasons may not be harmed. However, José shared his concerns about the future of his own orchard by revealing that in Santa Ana, located approximately twenty minutes away, hardly any orchards remain due to the lack of water for agricultural or even domestic use. Also, citrus fruit yields were still lower than normal in northwestern Mexico in the winter and spring 2014 and 2015 due to damage the trees sustained in 2013. Declines in fruit production are understood by farmers in San Ignacio to be an effect of drought; new studies also point to slower tree growth for up to four years from the time of the drought, and trees may also not recover because with climate change, another drought is increasingly likely to hit within that time period (Anderegg et al. 2015). This underscores the importance of studying the longer term impacts climate-related events have on agricultural livelihoods and of engaging with policy-makers to develop community-based adaptation programs that cushion the effects of these events in part by encouraging agrobiodiversity.

A newer strategy of this farming household to cope with climate effects (such as the loss of their citrus crops) and economic volatility is to rent out part of their orchard (to people from Nogales city) during the mild months of March through May. The orchard is equipped with a lean-to, makeshift stove, old sofas, table, and chairs for weekend and holiday parties; some even pitch tents and camp there overnight. The paying guests tend to invite José (who works in the orchard as they party) to eat with them when they barbecue meat, an added economic incentive. Catering to urban residents may become a more common strategy for farming households globally where urban areas are expanding and almost approaching agricultural areas and where climate is affecting the viability of agriculture.

Angela’s younger son often works with his uncle in the orchard and is likely to take it over in the future. He is also a skilled mechanic and welder who also works stints in local assembly plants and mines. None of these local employment options produce secure or sufficient income, even when combined, thus the family orchard remains an important source of income and food security. In March 2016, he was experimenting by planting different varieties of oak trees (*Quercus*), hoping to produce saplings out of cuttings taken from trees in the main plaza of San Ignacio that he could sell.

In another household, Celia decried the poor harvests in her husband’s orchard in 2012 and 2013 that were due to frost and insufficient rainfall. Celia stated that in other fields in and around San Ignacio, quince fruit harvests were even worse and that she and her son produced little quince paste. Her son now grows garlic and onions in addition to quince. However, in 2010, concerned about lower fruit production in his orchard, he also began to buy agricultural inputs like fertilizer and pesticides wholesale to sell to area farmers. This case and the case of José who rents out his orchard show a diversification in farm incomes directly related to climate and rainfall variability. Income diversification has been documented for other rural areas in Mexico and has been linked to economic stressors and to shifts in agricultural policy away from small-scale agriculture and toward support of large-scale, commercial production (De Janvry and Sadoulet 2001; Buechler 2002). In San Ignacio, economic and more recent climatic and water-related stressors coalesced to promote adaptation in the form of income diversification. Due to the larger size of their landholdings, men have more options than women to diversify their incomes through activities undertaken within their communities; also, in this and other U.S.-Mexico border communities, men migrate to the U.S. alone whereas women, particularly those with children, are less flexible and generally migrate only with family members (Segura and Zavella 2007).

Olives, cultivated in home gardens and in larger quantities around fruit orchard peripheries, have been affected for many years by the olive fruit fly (*Bactrocera oleae*); this pest has also affected other major olive-producing regions. Men and women increasingly purchase olives from vendors who buy from olive producers as far as 137 km away. Olive and olive oil production in San Ignacio is also declining due to lower tree replacement rates. If the drought worsens and tree deaths become more widespread, purchasing olives from other communities may no longer be a feasible adaptation strategy.

Challenges such as greater temperature and rainfall variability and increasing pest attacks are common to an increasing number of regions globally. Strategies employed by farmers in Sonora’s small production spaces offer a glimpse into local responses to environmental change; a better understanding of these could provide insight into programs and policies that would support and augment these local adaptive responses as well as assist other farmers facing similar climate and water-related challenges.

### Building on local adaptation strategies

In addition to facilitating analysis of gender differences in production spaces and differential access to assistance, an FPE framework also brings into focus strategies that could build on local, gendered adaptive responses. Women’s and men’s adaptation activities in San Ignacio could be enhanced by projects that confer greater income and expand access to credit, technologies and knowledge. In order to address issues of adverse inclusion and the inequities associated with power differentials between small and larger producers, credit could be offered by government agencies (perhaps for use in revolving loans) that would be solely for small producers and agricultural extension could be offered to female and male farmers that would be tailored to their particular, smaller production spaces. Community-based adaptation groups could be formed among new or existing community groups and linked to government agencies like SAGARPA in order to build on individual’s strategies. Projects would ideally focus on reducing water use and have carbon mitigation potential. There is a growing realization of the carbon mitigation potential of fruit trees (Verchot et al. 2007; Matocha et al. 2012). Policies tied to such mitigation projects should address larger scale barriers such as the lack of support in Sonora for fruit tree production. Trees absorb carbon dioxide during photosynthesis, store this carbon in their tissues and soils, and sequester it from the atmosphere (Kimball et al. 2007). Studies in semi-arid regions such as Italy on olive groves as well as peach and other orchards have indicated that the resilience of orchard growers can be enhanced if sustainable practices are taught to farmers (Montanaro et al. 2012; Palese et al. 2013). These practices include using greywater for irrigation as some women in San Ignacio’s home gardens already take advantage of. Extension and subsidies for greywater systems could enable more women to use greywater, or enable those women who currently utilize greywater with simple and often leaky systems, to irrigate with it more efficiently, extending the use of increasingly scarce water resources. Non-profit or government programs to teach farmers about certain methods to ensure healthier plants better able to withstand climate variability are further options. Such methods include increasing carbon inputs to the soil (mainly compost, manure, biochar^1^ and once annual pruning replacing chemical fertilizers) and limiting soil respiration through no tillage and minimal grass mowing (to largely replace herbicidal weed control and to increase soil fertility) (Fiore 2014).^2^

## Conclusions

Longer term ethnographic research with women and men in San Ignacio revealed that small-scale agricultural producers have adapted in the face of adverse environmental conditions. This study points to the need for researchers, policy-makers, and activists to give greater weight to local women’s and men’s adaptation-related capacities and initiatives. These are based on women and men’s lived experiences with agriculture. These experiences differ by gender in San Ignacio; this is partly because gender norms structure the location and size of agricultural landholdings and because social class and type of agricultural production affects access to government assistance. Unlike livestock ranchers in the state of Sonora who receive, for example, subsidized bales of high water-consuming alfalfa (Buechler 2015), both male and female fruit and vegetable farmers in San Ignacio, Sonora obtain far less government assistance. This occurs despite the fact that such production is beginning to be recognized globally as offering climate change mitigation potential that can be maximized if sustainable practices are taught to farmers. Such forms of agriculture also use land and water resources in an efficient manner translating into more sustainable adaptation strategies, especially if they are part of a diversified cropping pattern. Diversified production also provides some protection against climate and water-related risks and offers nutritious food for human consumption. Although all fruit producers in San Ignacio receive little government support, female farmers in kitchen gardens receive even less agriculture-related government assistance than male farmers with small orchards.

In the absence of government assistance due to the neoliberal context in which agriculture is inserted in Mexico and to adverse inclusion of smallholders in government crop support, credit and extension programs, women and men in San Ignacio have engaged in creative and dynamic adaptation strategies to confront the challenges of environmental change. The spaces that women and men farm are different due to gendered land ownership, and the type of water available to each space is also distinct. The spaces farmed affect the adaptation strategies women and men develop. In the agriculture-devoted spaces of their home compounds, women engage in experimentation including the re-introduction of fruit crops. This strategy is a longer term one, focused on adapting to present and future changes, such as increased pest attacks and climate variability. Women practice flexible input sourcing too (such as with the purchase of olives from areas not affected by a pest attack) in order to adapt. Women also increase home garden biodiversity, a practice that provides households with subsistence crops and income even in years of pest infestation and/or weather extremes. The spillover effects in men’s larger plots include an overall emphasis on multi-cropping rather than monocropping.

Women innovate with respect to irrigation water sources, such as by reusing water to make a scarce resource stretch further and by combining more than one source. These strategies hold important lessons for other regions experiencing similar water and climate challenges including more frequent and more prolonged periods of drought, greater rainfall variability, and increased evaporation of existing rainfall due to higher temperatures. Women’s home garden production options and their adaptive capacities are more limited than those of male fruit farmers, however, due to the fact that the water supply women depend on is shared with the domestic water supply limiting the volume available for agriculture.

Men with small land parcels have adapted in myriad ways such as through crop diversification (which women also engage in within home garden production) and sharing crops with wildlife pushed out of their habitats. Crop diversification provides some insurance against extreme weather events like frost and drought. Long-term drought, though, will likely require external assistance to build on men’s current initiatives such as the production of drought-tolerant crops like the nopal cactus. Men are also engaging in adaptation strategies like adding on-farm and non-farm income sources and renting orchards to urban residents. Those strategies are more available to men because of men’s larger agricultural landholding sizes. Non-farm employment in this area (and in many areas surrounding agricultural communities worldwide) is poorly remunerated and unstable but can reduce risk associated with shorter term crop loss or longer term yield reductions. Migration can also cushion environmental effects on agriculture and is engaged in by men more frequently than women.

Both women and men’s adaptation initiatives are often hampered by structural inequalities such as those related to social class like inequities between small and large landowners, by higher transaction costs for smallholder men related to time and money spent in travel to government offices to do necessary paperwork, and by a general lack of attention to small-scale fruit and vegetable producers and to fruit and vegetable production compared with livestock and fodder production. These inequalities, which lead to adverse inclusion, can be reduced through collaborations between local peoples and government or non-governmental programs that aim to provide services like credit and extension to small farmers, facilitate the completion of paperwork such as by offering online services for small farmers and aim to support vegetable and fruit production statewide. In addition to assisting community members, such initiatives, if combined with mitigation projects, could slow global warming.

The combined use of adaptation and FPE frameworks in addition to the concept of adverse inclusion helped shape a focus on socially differentiated (by gender and social class), grassroots initiatives developed in response to a dynamic and often hostile environmental, economic, and policy context. This study builds on FPE that examines social divisions of labor in natural resource management by stressing the importance of the role of place or location in these divisions. This study also expands on research that connects climate change, water resource pressures, and agriculture by adding a focus on who is farming what type of land within agricultural communities and what their responses to a changing environment are.

An FPE approach helped reveal that officials and decision-makers need to place priority on agricultural livelihoods carried out in spaces such as home compounds and small orchards and on the social, economic, and political positioning of women and men who farm there under increasingly volatile environmental conditions. The strategies these women and men develop over time, some of which are the same and some of which differ by gender, can provide keys to revealing locally appropriate, dynamic adaptation and mitigation activities and to the formulation of locally and regionally appropriate programs and policies that build on these grassroots initiatives.

## Footnotes

1 Biochar is charcoal produced from plant matter and stored in soil to remove atmospheric carbon dioxide.

2 Using these methods, resulting higher emissions (via higher carbon inputs) are usually compensated for by increased soil carbon storage.
